# Supplementary material for: A stimulus‐contingent positive feedback loop enables IFN‐β dose‐dependent activation of pro‐inflammatory genes
Source: Mol Syst Biol. 2023 Mar 17;19(5):e11294. doi: 10.15252/msb.202211294 (PMC10167482; doi:10.15252/msb.202211294)
Supplement: Supplementary file 1 — Expanded View Figures PDF [file MSB-19-e11294-s011.pdf]

Expanded View Figures

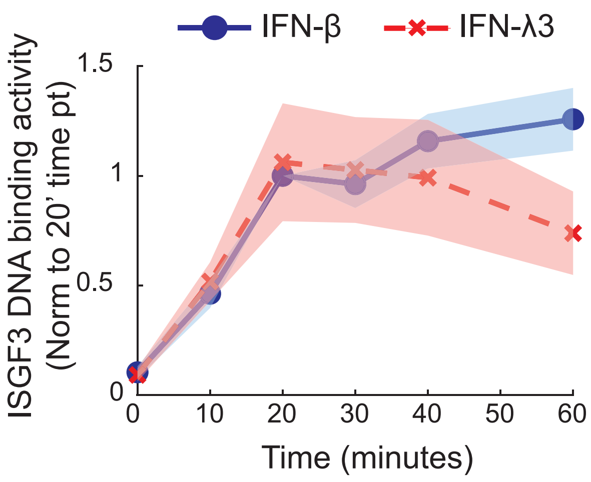

Figure EV1. IFN-β and IFN-λ3 show comparable ISGF3 activation at the selected concentrations.

Quantified ISGF3 activity upon IFN-β (10 U/ml, blue) and IFN-λ3 (100 ng/ml, red) stimulation measured by an electrophoretic mobility shift assay (EMSA). Data are a subset of the time course data from Fig 2 indicating the ISGF3 activity in the first hour (i.e., activation phase).

Source data are available online for this figure.

Figure EV2. Applying the adapted IFN signaling dynamical network model to assess STAT2- and IRF9-feedback loop-dependent temporal signaling dynamics.

Using the adapted IFN signaling model (Fig 3), simulations predict ISGF3 binding activity, nuclear and cytoplasmic active and total protein species, and mRNA species when positive feedback loops for STAT2 (yellow line), IRF9 (purple line), and STAT1 (green line) are eliminated or not (thick blue line) during IFN-β stimulation.

© 2023 The Authors

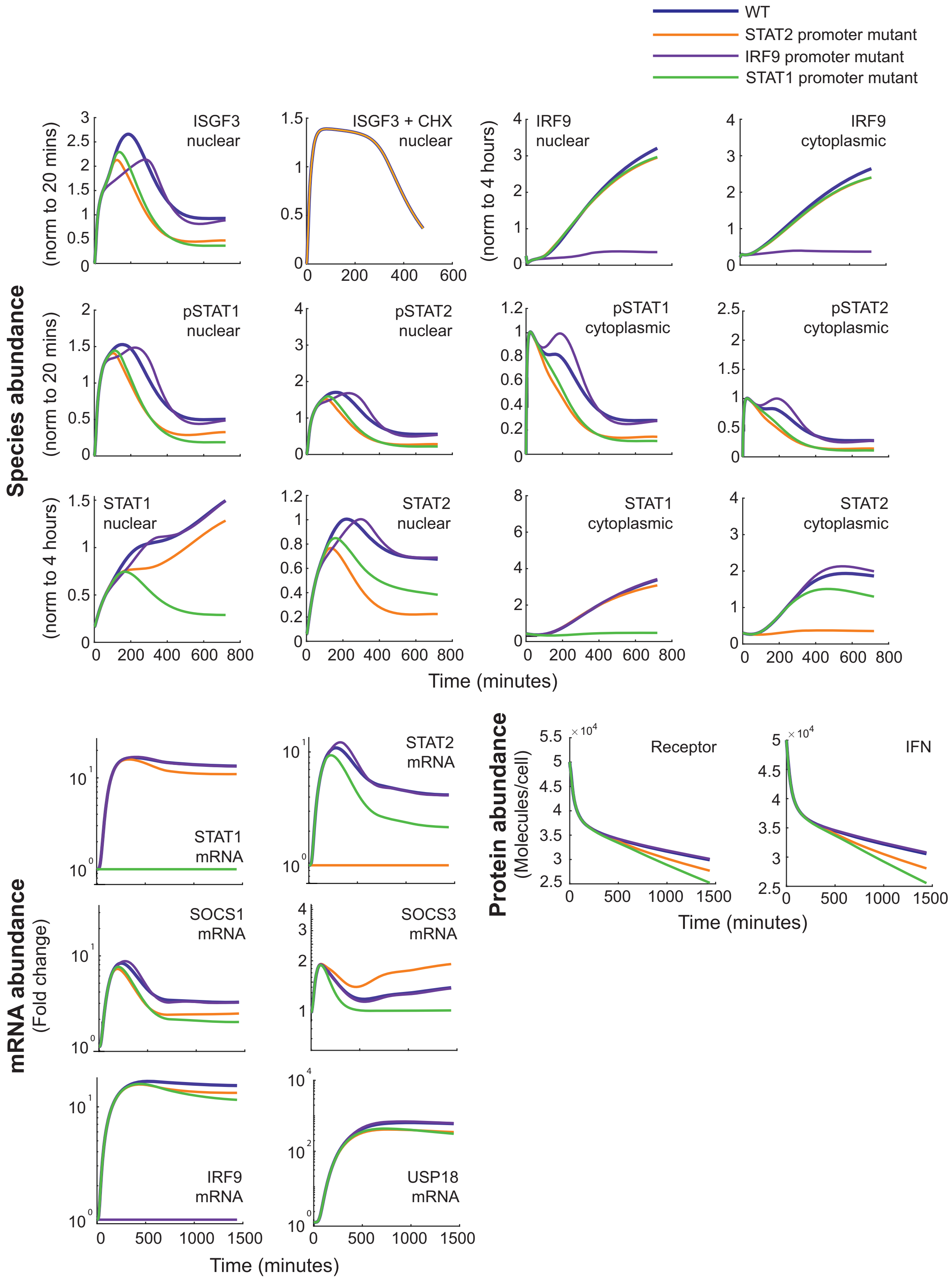
WT
STAT2 promoter mutant
IRF9 promoter mutant
STAT1 promoter mutant
Species abundance
(norm to 20 mins)
(norm to 4 hours)
ISGF3 nuclear
ISGF3 + CHX nuclear
IRF9 nuclear
IRF9 cytoplasmic
pSTAT1 nuclear
pSTAT2 nuclear
pSTAT1 cytoplasmic
pSTAT2 cytoplasmic
STAT1 nuclear
STAT2 nuclear
STAT1 cytoplasmic
STAT2 cytoplasmic
Time (minutes)
mRNA abundance
(Fold change)
STAT1 mRNA
STAT2 mRNA
SOCS1 mRNA
SOCS3 mRNA
IRF9 mRNA
USP18 mRNA
Protein abundance
(Molecules/cell)
Receptor
IFN

Figure EV2.

Figure EV3. CRISPR-Cas9 gene editing effectively mutates ISGF3 binding motifs in STAT2 and IRF9 gene promoters.

A Nucleotide sequences of the promoter regions of murine IRF9 (ENSMUSG00000002325) and STAT2 (ENSMUSG00000040033) genes containing an ISGF3 binding motif. Distance from TSS was selected based on the locus of the IRF9 transcript (ENSMUST00000138037.2) and STAT2 transcript (ENSMUST00000085708.3). Data of the IRF9 promoter and STAT2 promoter regions from cDNA of IRF9 promoter and STAT2 promoter mutant MLE-12 lung epithelial cells measured using Sanger sequencing. Alterations near the targeted ISGF3 binding motif are indicated in red text. Data are from two biological replicates.

B Pie charts of unique DNA sequences of the promoter regions of IRF9 and STAT2 genes, indicating the percentage of DNA sequences of the promoter region of IRF9 and STAT2 from IRF9 promoter and STAT2 promoter mutant cells, respectively, measured using amplicon sequencing. The WT variant compromised 21% of the total IRF9 DNA and 9% of the STAT2 DNA, with mutant variants found in the remaining 79 and 91%, respectively.

Source data are available online for this figure.

 © 2023 The Authors

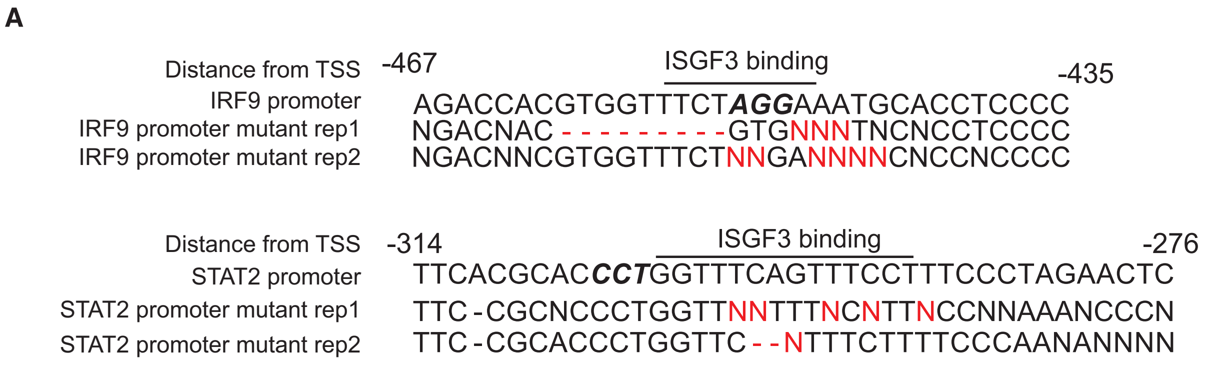

B

Percentage of IRF9 promoter sequcences

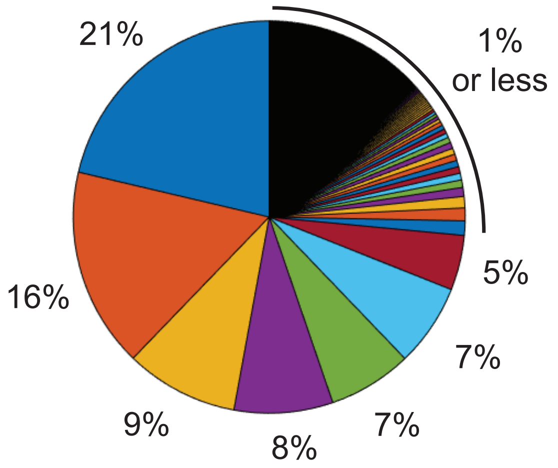

%	Sequence
21	AGACCACGTGGTTTCTAGGAAATGCACCTCCCC
16	TGACACAGAGGATGGGGTT
9	AGACCACGTGGTCTAGGAAATGCACCTCCCC
8	AGACCACGTGGAAATGCACCTCCCC
7	GCCCATGTGTCAGAAAAGAAGGGACTCTCAAGCCCC
7	AGACTCATGGCCACCCTCT
5	AGACCACGTGGTTCTAGGAAATGCACCTCCCC

Percentage of STAT2 promoter sequcences

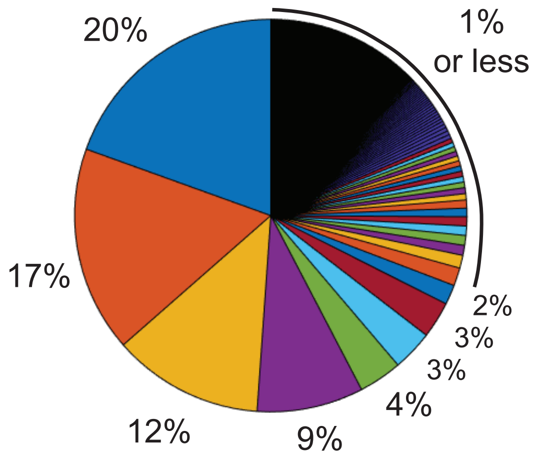

%	Sequence
20	TTCACGCACCCTGGTTCAGTTTCCTTTCCCTAGAACTC
17	TTCACGCACCCTGTTCAGTTTCCTTTCCCTAGAACTC
12	TTCACGCACCCTGGTTTTCAGTTTCCTTTCCCTAGAACTC
9	TTCACGCACCCTGGTTTCAGTTTCCTTTCCCTAGAACTC
4	TTCACGCACCCTGGTCAGTTTCCTTTCCCTAGAACTC
3	TTCACGCACCCTGGTTTCCTTTCCCTAGAACTC
3	TTCACGCACCCTTCAGTTTCCTTTCCCTAGAACTC

Figure EV3.

© 2023 The Authors
