# Supplementary material for: A stimulus‐contingent positive feedback loop enables IFN‐β dose‐dependent activation of pro‐inflammatory genes
Source: Mol Syst Biol. 2023 Mar 17;19(5):e11294. doi: 10.15252/msb.202211294 (PMC10167482; doi:10.15252/msb.202211294)
Supplement: Supplementary file 10 — Source Data for Figure 3 [file MSB-19-e11294-s007.zip › Source Data for Figure 3/3C/Souce Data Fig 3 cyto phospho and total STAT1 Western.pdf]

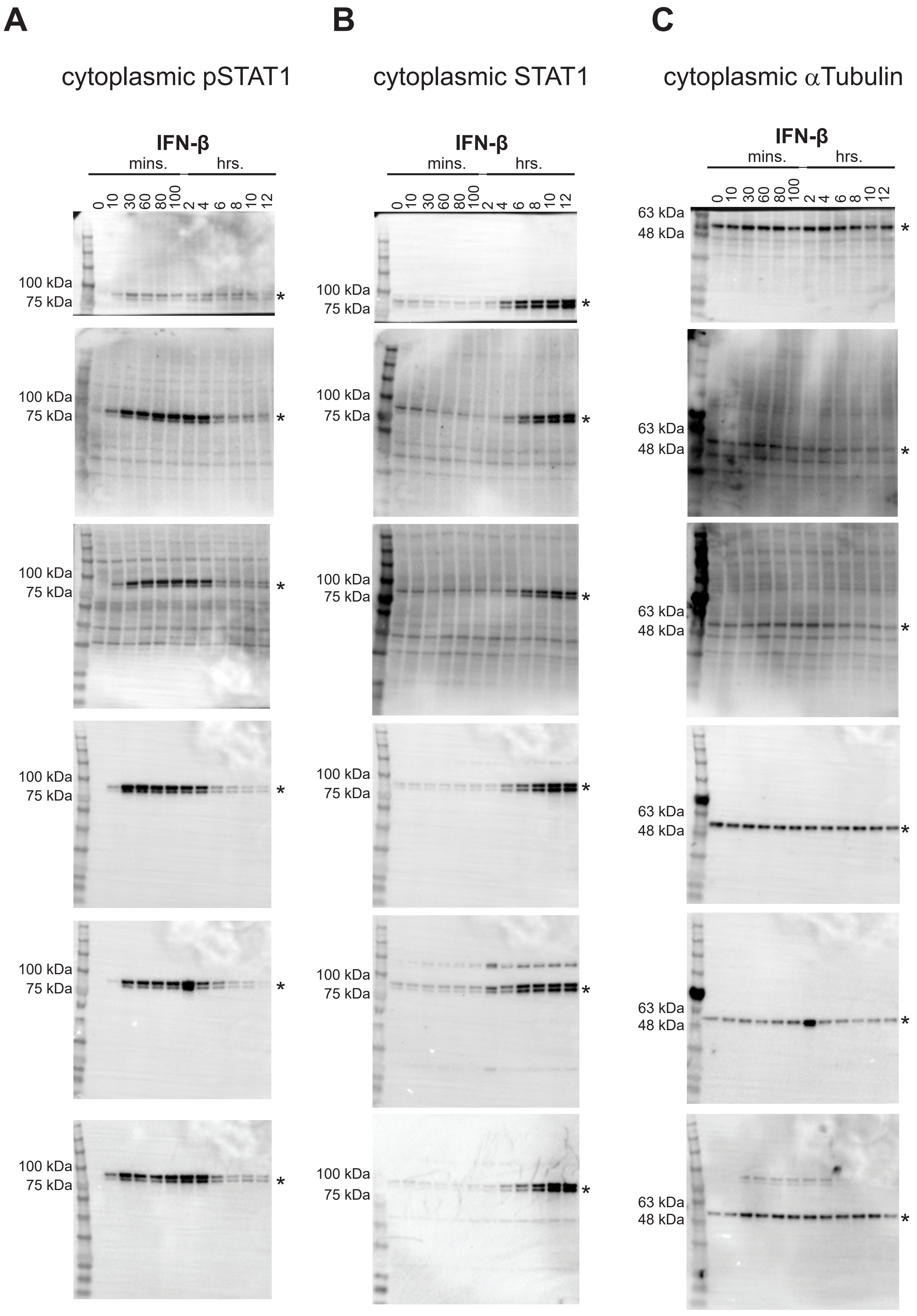

Source Data Figure S5: Characterization of cytoplasmic active and total STAT1 temporal dynamics (supports Figure 3C). Immunoblot data of (A) phosphorylated STAT1 and (B) total STAT1 compared to the (C) constitutive αTubulin loading control from cytoplasmic extracts collected during 10 U/ml IFN-β stimulation. Asterisk indicates band at expected electrophoretic mobility. Six independent experiments are shown.
