# Supplementary material for: A stimulus‐contingent positive feedback loop enables IFN‐β dose‐dependent activation of pro‐inflammatory genes
Source: Mol Syst Biol. 2023 Mar 17;19(5):e11294. doi: 10.15252/msb.202211294 (PMC10167482; doi:10.15252/msb.202211294)
Supplement: Supplementary file 10 — Source Data for Figure 3 [file MSB-19-e11294-s007.zip › Source Data for Figure 3/3C/Souce Data Fig 3 nuclear IRF9 Western.pdf]

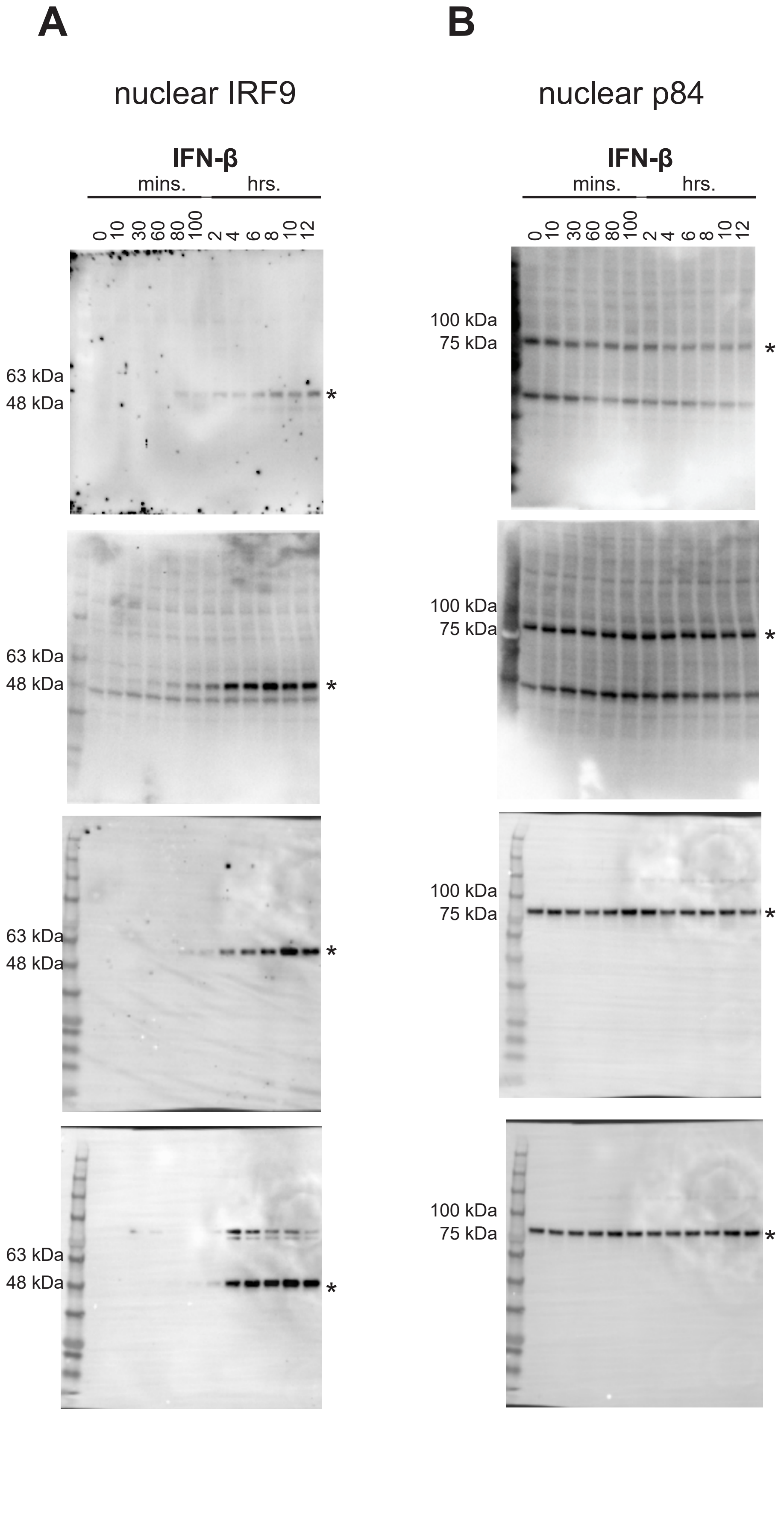

Source Data Figure S4: Characterization of nuclear total IRF9 temporal dynamics (supports Figure 3C). Immunoblot data of (A) total IRF9 compared to the (B) constitutive nuclear matrix protein p84 loading control from nuclear extracts collected during 10 U/ml IFN-β stimulation. Asterisk indicates band at expected electrophoretic mobility. Four independent experiments are shown.
