# Supplementary material for: A stimulus‐contingent positive feedback loop enables IFN‐β dose‐dependent activation of pro‐inflammatory genes
Source: Mol Syst Biol. 2023 Mar 17;19(5):e11294. doi: 10.15252/msb.202211294 (PMC10167482; doi:10.15252/msb.202211294)
Supplement: Supplementary file 10 — Source Data for Figure 3 [file MSB-19-e11294-s007.zip › Source Data for Figure 3/3C/Source Data Fig 3 EMSA CHX.pdf]

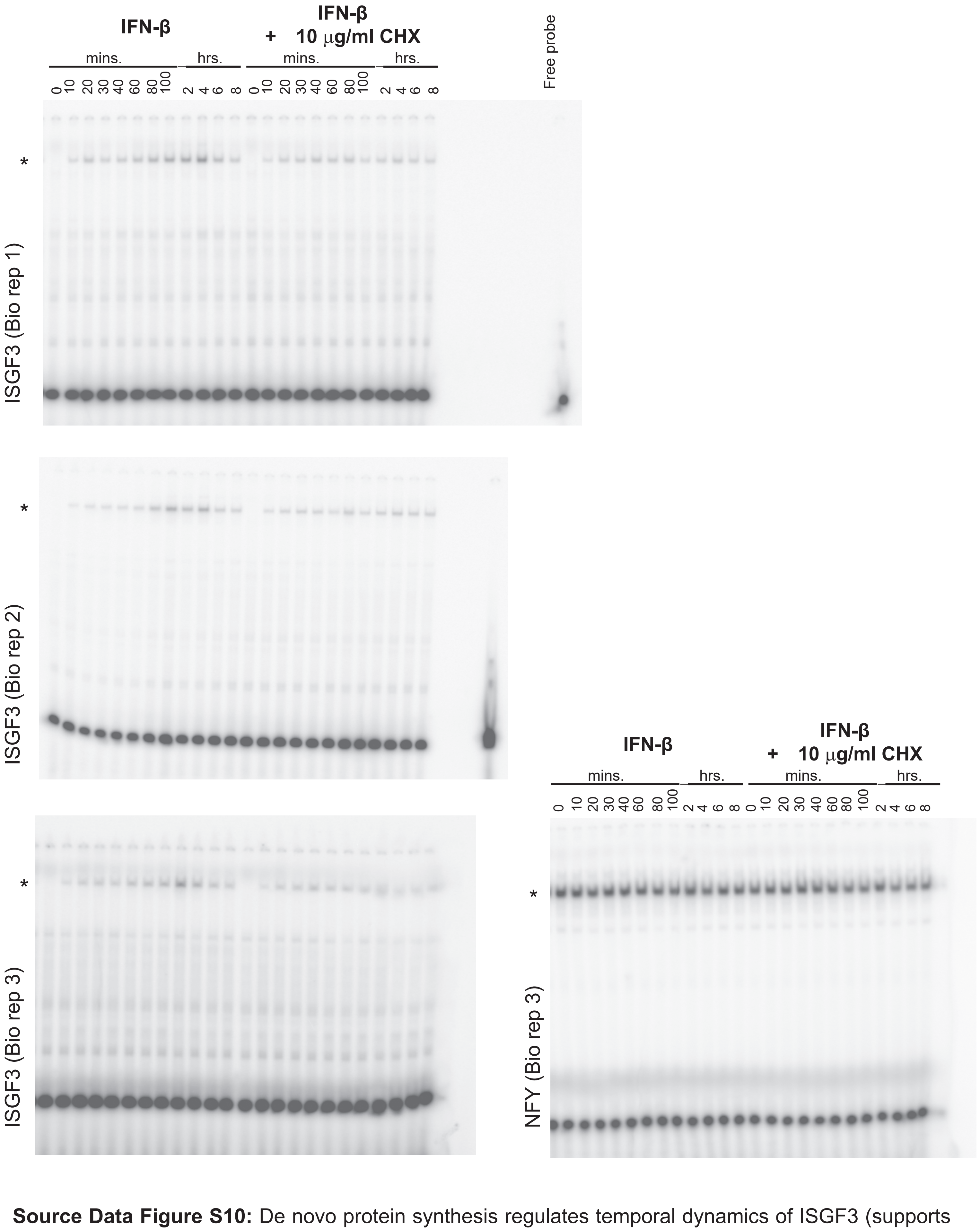

Source Data Figure S10: De novo protein synthesis regulates temporal dynamics of ISGF3 (supports Figure 5A). ISGF3 and constitutive NFY activity temporal dynamics during stimulation with 10 U/ml IFN-β with or without 10 μg/ml cycloheximide (CHX), an inhibitor of protein synthesis, measured by EMSA. Asterisk indicates band at expected electrophoretic mobility. Three independent experiments are shown.
