# Supplementary material for: A stimulus‐contingent positive feedback loop enables IFN‐β dose‐dependent activation of pro‐inflammatory genes
Source: Mol Syst Biol. 2023 Mar 17;19(5):e11294. doi: 10.15252/msb.202211294 (PMC10167482; doi:10.15252/msb.202211294)
Supplement: Supplementary file 10 — Source Data for Figure 3 [file MSB-19-e11294-s007.zip › Source Data for Figure 3/3C/Source Data Fig 3 EMSA.pdf]

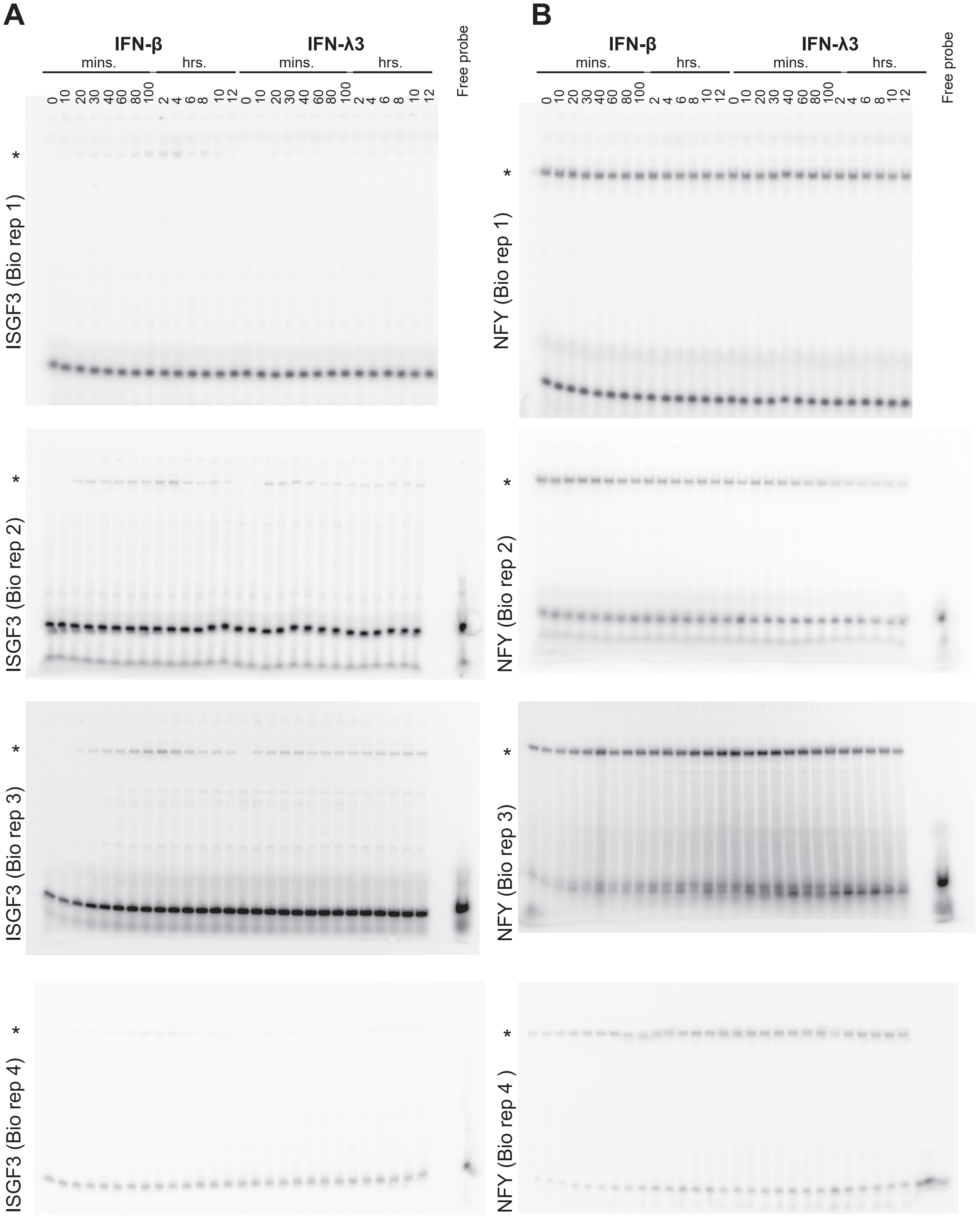

Source Data Figure S1: Stimulus-specific temporal dynamics of ISGF3 (supports Figure 2A and B). (A) ISGF3 activity revealed by an EMSA using an ISRE oligo probe and nuclear extracts prepared from MLE-12 cells at indicated time points of IFN-β (10 U/ml) and IFN-λ3 (100 ng/ml) stimulation. (B) Constitutive transcription factor NFY was used as a loading control. Asterisk indicates band at expected electrophoretic mobility. Four independent experiments are shown.
