# Supplementary material for: A stimulus‐contingent positive feedback loop enables IFN‐β dose‐dependent activation of pro‐inflammatory genes
Source: Mol Syst Biol. 2023 Mar 17;19(5):e11294. doi: 10.15252/msb.202211294 (PMC10167482; doi:10.15252/msb.202211294)
Supplement: Supplementary file 10 — Source Data for Figure 3 [file MSB-19-e11294-s007.zip › Source Data for Figure 3/3C/Souce Data Fig 3 cyto IRF9 Western.pdf]

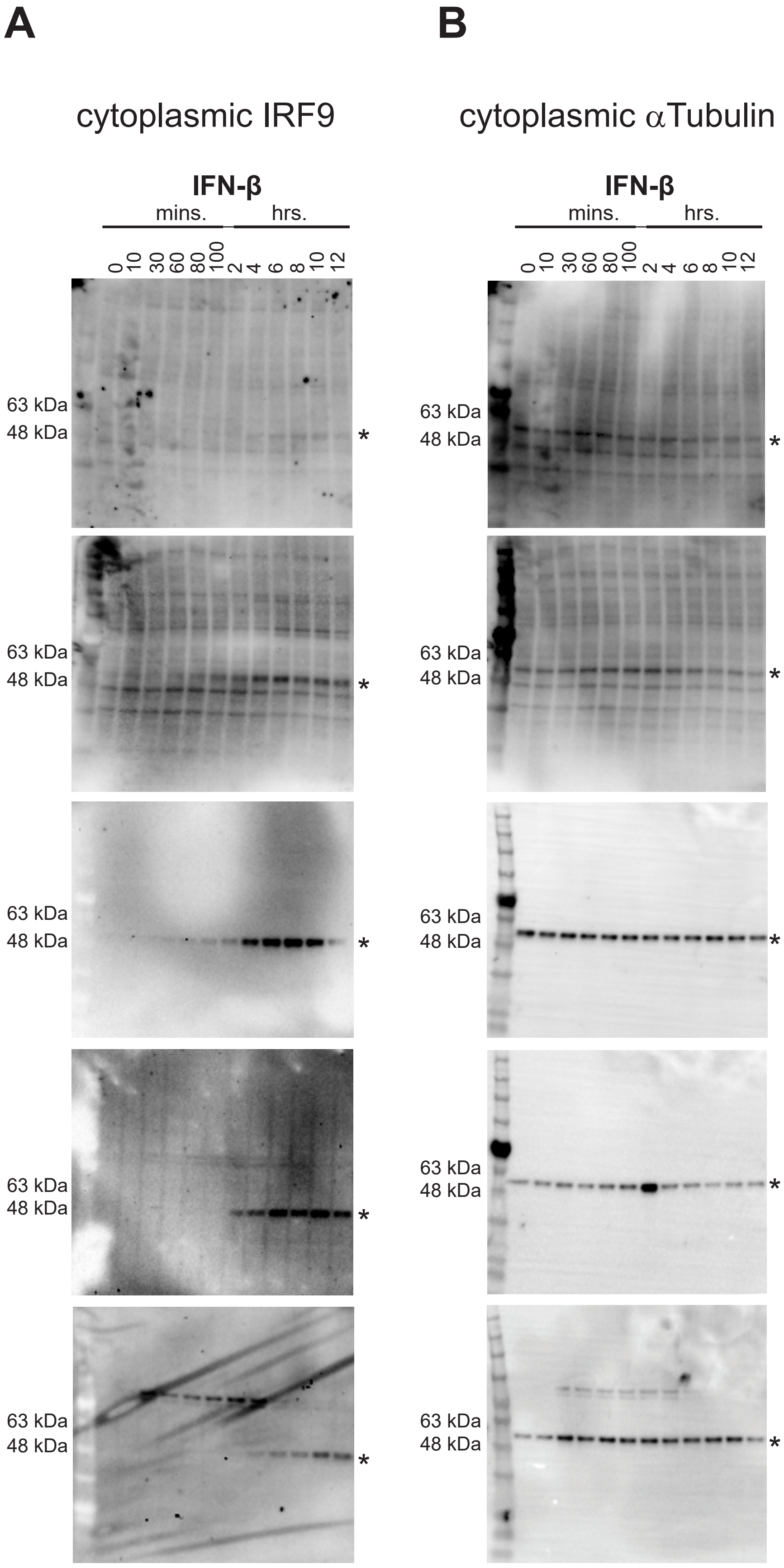

Source Data Figure S7: Characterization of cytoplasmic total IRF9 temporal dynamics (supports Figure 3C). Immunoblot data of (A) total IRF9 compared to the (B) constitutive αTubulin loading control from cytoplasmic extracts collected during 10 U/ml IFN-β stimulation. Asterisk indicates band at expected electrophoretic mobility. Five independent experiments are shown.
