# Supplementary material for: A stimulus‐contingent positive feedback loop enables IFN‐β dose‐dependent activation of pro‐inflammatory genes
Source: Mol Syst Biol. 2023 Mar 17;19(5):e11294. doi: 10.15252/msb.202211294 (PMC10167482; doi:10.15252/msb.202211294)
Supplement: Supplementary file 11 — Source Data for Figure 4 [file MSB-19-e11294-s006.zip › Source Data for Figure 4/4F/Source Data Fig 4 EMSA pulse.pdf]

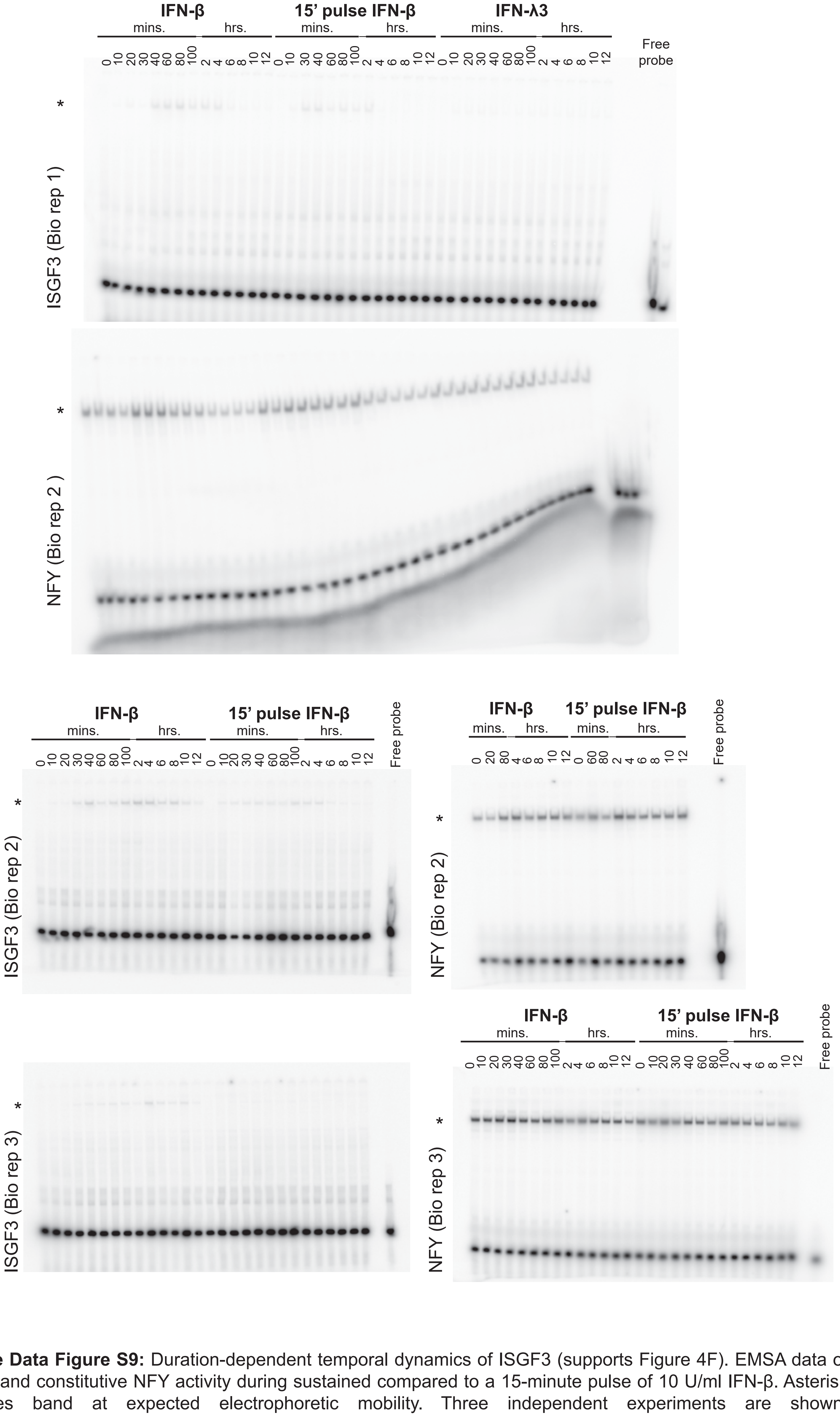


**Source Data Figure S9:** Duration-dependent temporal dynamics of ISGF3 (supports Figure 4F). EMSA data of ISGF3 and constitutive NFY activity during sustained compared to a 15-minute pulse of 10 U/ml IFN-β. Asterisk indicates band at expected electrophoretic mobility. Three independent experiments are shown.
