# Supplementary material for: A stimulus‐contingent positive feedback loop enables IFN‐β dose‐dependent activation of pro‐inflammatory genes
Source: Mol Syst Biol. 2023 Mar 17;19(5):e11294. doi: 10.15252/msb.202211294 (PMC10167482; doi:10.15252/msb.202211294)
Supplement: Supplementary file 11 — Source Data for Figure 4 [file MSB-19-e11294-s006.zip › Source Data for Figure 4/4C-D/Source Data Fig 4 EMSA dose curve.pdf]

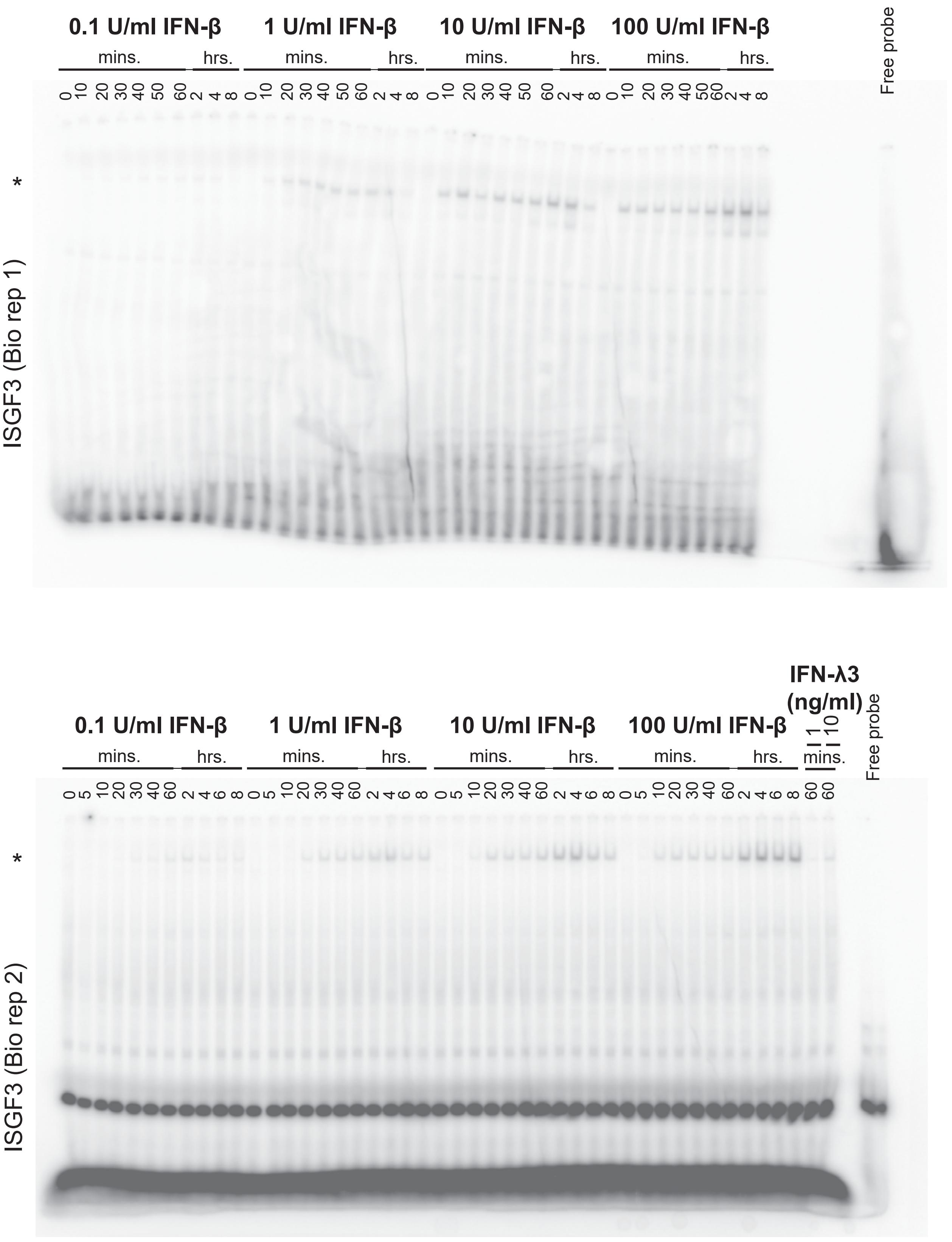

Source Data Figure S8: Dose-dependent temporal dynamics of ISGF3 (supports Figure 4C and 4D). ISGF3 activity revealed by an EMSA during 10 U/ml, 1 U/ml, and 0.1 U/ml IFN-β stimulation. Asterisk indicates band at expected electrophoretic mobility. Two independent experiments are shown.
