# Supplementary material for: A stimulus‐contingent positive feedback loop enables IFN‐β dose‐dependent activation of pro‐inflammatory genes
Source: Mol Syst Biol. 2023 Mar 17;19(5):e11294. doi: 10.15252/msb.202211294 (PMC10167482; doi:10.15252/msb.202211294)
Supplement: Supplementary file 12 — Source Data for Figure 5 [file MSB-19-e11294-s001.zip › Source Data for Figure 5/5F/Source Data Fig 5 EMSA mutants.pdf]

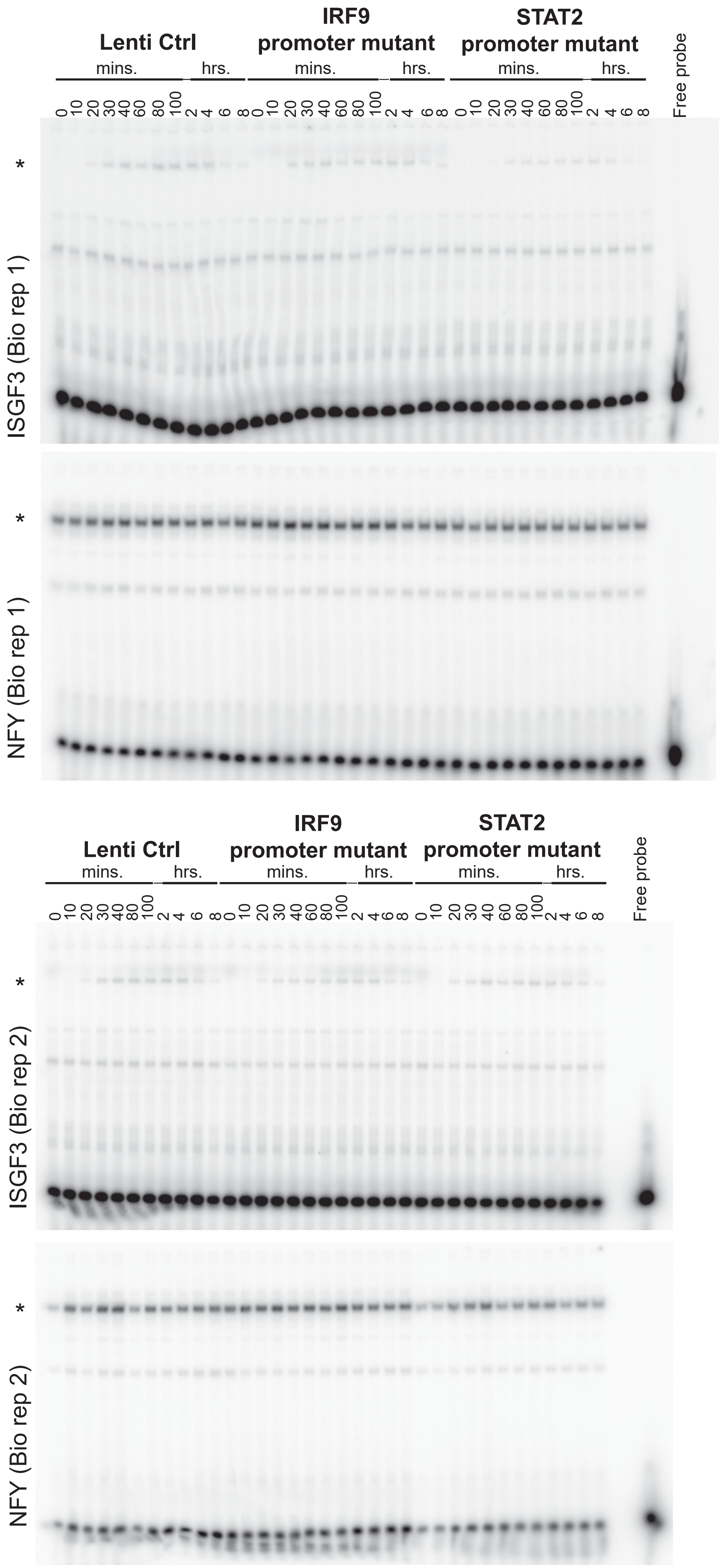

Source Data Figure S11: STAT2- and IRF9-feedback-loop-dependent ISGF3 temporal dynamics (supports Figure 5F). EMSA data of the temporal dynamics of ISGF3 activity in MLE-12 lung epithelial cells with mutations in the STAT2 promoter, IRF9 promoter, or a lentiviral control when stimulated with 2.77 U/ml IFN-β compared to the constitutive NFY control. Asterisk indicates band at expected electrophoretic mobility. Two independent experiments are shown.
