# Supplementary material for: A stimulus‐contingent positive feedback loop enables IFN‐β dose‐dependent activation of pro‐inflammatory genes
Source: Mol Syst Biol. 2023 Mar 17;19(5):e11294. doi: 10.15252/msb.202211294 (PMC10167482; doi:10.15252/msb.202211294)
Supplement: Supplementary file 12 — Source Data for Figure 5 [file MSB-19-e11294-s001.zip › Source Data for Figure 5/5C/Souce Data Fig 5 nuclear phospho and total STAT2 Western.pdf]

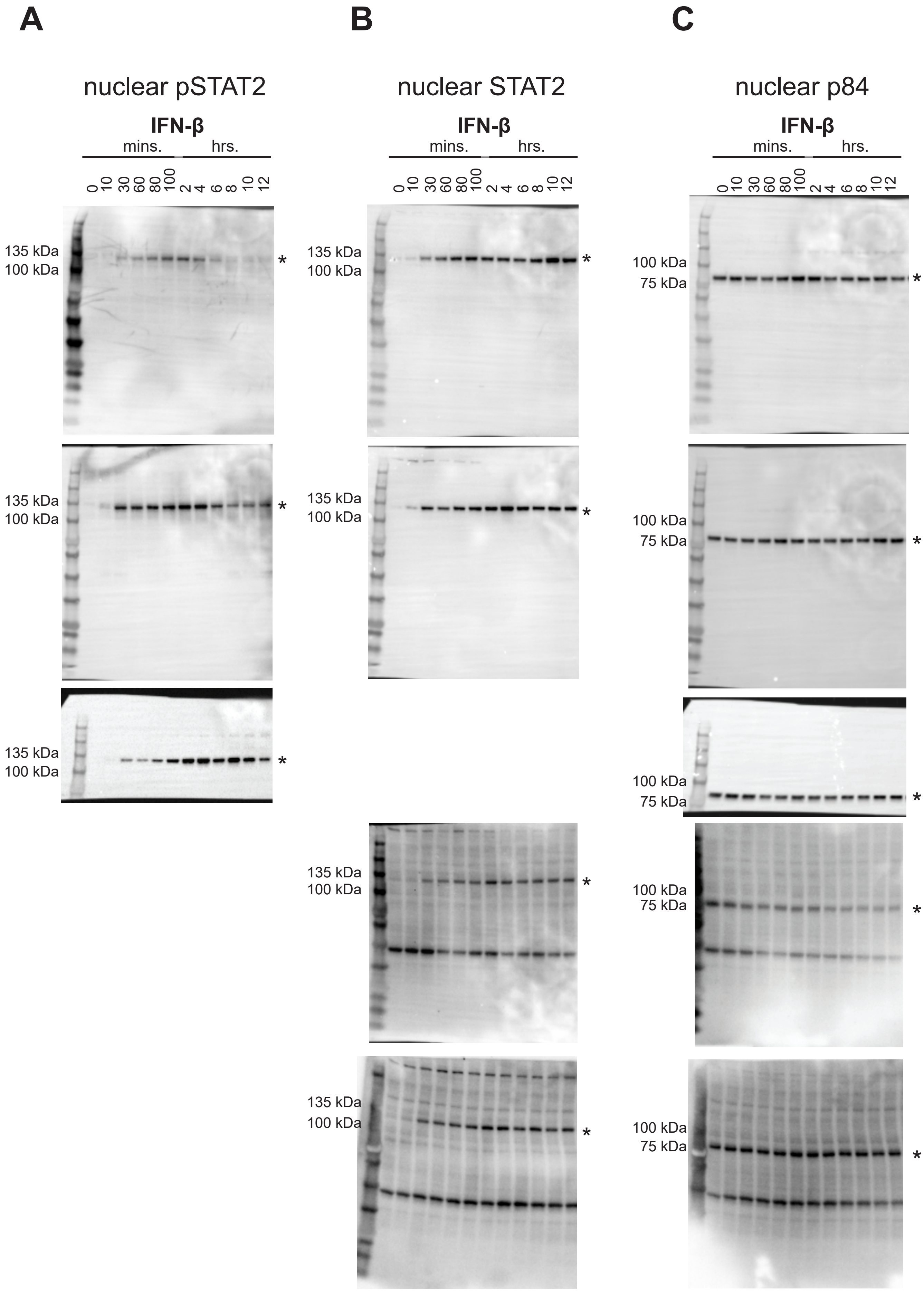

Source Data Figure S3: Characterization of nuclear active and total STAT2 temporal dynamics (supports Figure 3C). Immunoblot data of (A) phosphorylated STAT2 and (B) total STAT2 compared to the (C) constitutive nuclear matrix protein p84 loading control from nuclear extracts collected during 10 U/ml IFN-β stimulation. Asterisk indicates band at expected electrophoretic mobility. Five independent experiments are shown.
