# Supplementary material for: A stimulus‐contingent positive feedback loop enables IFN‐β dose‐dependent activation of pro‐inflammatory genes
Source: Mol Syst Biol. 2023 Mar 17;19(5):e11294. doi: 10.15252/msb.202211294 (PMC10167482; doi:10.15252/msb.202211294)
Supplement: Supplementary file 12 — Source Data for Figure 5 [file MSB-19-e11294-s001.zip › Source Data for Figure 5/5C/Souce Data Fig 5 nuclear phospho and total STAT1 Western.pdf]

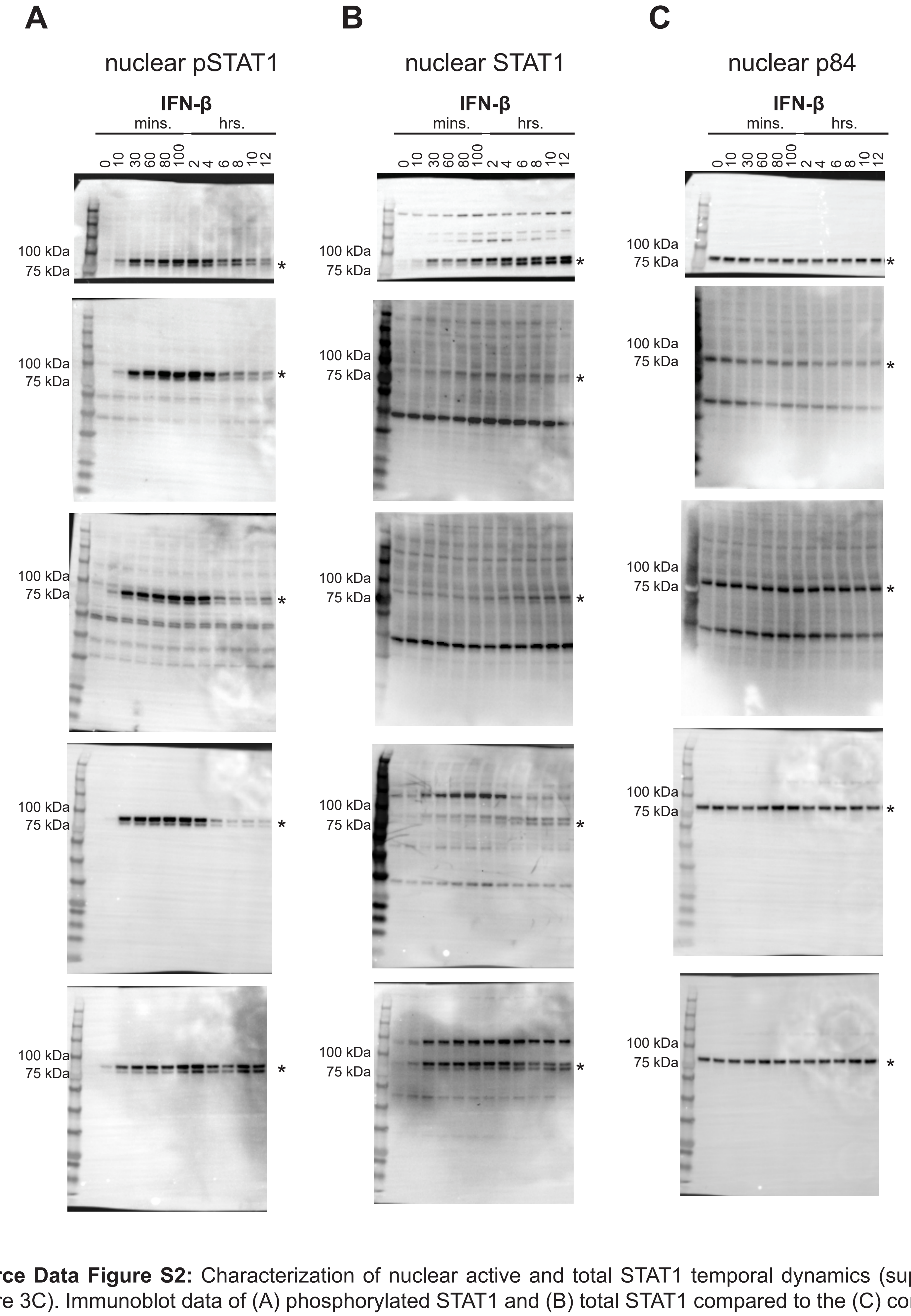

Source Data Figure S2: Characterization of nuclear active and total STAT1 temporal dynamics (supports Figure 3C). Immunoblot data of (A) phosphorylated STAT1 and (B) total STAT1 compared to the (C) constitutive nuclear matrix protein p84 loading control from nuclear extracts collected during 10 U/ml IFN-β stimulation. Asterisk indicates band at expected electrophoretic mobility. Five independent experiments are shown.
