# Supplementary material for: A stimulus‐contingent positive feedback loop enables IFN‐β dose‐dependent activation of pro‐inflammatory genes
Source: Mol Syst Biol. 2023 Mar 17;19(5):e11294. doi: 10.15252/msb.202211294 (PMC10167482; doi:10.15252/msb.202211294)
Supplement: Supplementary file 13 [file MSB-19-e11294-s012.pdf]

molecular systems biology

A stimulus-contingent positive feedback loop enables IFN-β activation of pro-inflammatory genes

Catera Wilder, Diane Lefaudeux, Raisa Mathenge, Kensei Kishimoto, Alma Zuniga Munoz, Minh Nguyen, Aaron Meyer, Quen Cheng, and Alexander Hoffmann
DOI: 10.15252/msb.202211294

Corresponding author(s): Alexander Hoffmann (ahoffmann@ucla.edu)

Review Timeline:

Submission Date:	10th Aug 22
Editorial Decision:	20th Sep 22
Revision Received:	16th Jan 23
Editorial Decision:	9th Feb 23
Revision Received:	28th Feb 23
Accepted:	1st Mar 23

Editor: Maria Polychronidou

Transaction Report:

(Note: With the exception of the correction of typographical or spelling errors that could be a source of ambiguity, letters and reports are not edited. Depending on transfer agreements, referee reports obtained elsewhere may or may not be included in this compilation. Referee reports are anonymous unless the Referee chooses to sign their reports.)

1st Editorial Decision 20th Sep 2022

Thank you again for submitting your work to Molecular Systems Biology. We have now heard back from the three reviewers who agreed to evaluate your study. As you will see below, the reviewers think that the study is a relevant contribution to the field. However, they raise a series of concerns, which we would ask you to address in a revision.

The recommendations of the reviewers seem quite clear and I therefore see no need to repeat any of them here. All issues raised by the referees would need to be satisfactorily addressed. Please let me know in case you would like to discuss in further detail any of the issues raised, I would be happy to schedule a call.

Reviewer #1:

In this manuscript, Wilder and coworkers investigate differential gene responses induced by interferon (IFN)-beta vs. IFN-lambda. Experiments were performed in mouse lung epithelial cells (MLE-12). Time-resolved RNA-seq data showed that IFN-beta induced a strong, transient induction 4 to 8 h after stimulation of a large cluster of genes associated with cytokine production and immune response. The same genes showed a sustained accumulation with slower dynamics upon stimulation with IFN-lambda. Similar dynamics were observed by an electrophoretic mobility shift assay (EMSA) using an ISRE probe. A simple mathematical model demonstrated that the average expression profile upon IFN-beta and IFN-lambda stimulation could be explained by this ISRE binding data. A previously published mathematical model was re-fitted to time-resolved experimental data based on immunoblotting and EMSA experiments. Model simulations and experiments showed that for the high and transient gene expression, a high and sustained dose of IFN-beta as well as de novo protein synthesis was required. IFN-beta induced the expression of IRF9 and STAT2, comprising a positive feedback loop. By removing the ISRE sites of IRF9 and STAT2 via CRISPR-Cas9, this positive feedback loop was abolished and consequently the high and transient gene expression induced by IFN-beta was reduced.
The experimental data as wells as the mathematical modeling approaches are of high quality. The conclusions, although not completely unexpected, are supported by the data and clearly described. Dynamic control of IFN-induced gene response is a timely topic, given the importance of IFN signal transduction in antiviral responses.

Major comments:

1) Fig. 1D: The selection of genes was based on transcripts that are induced by IFN-beta. Are there transcripts that are only induced by IFN-lambda? If yes, a supplementary figure with these transcripts should be included.
2) Fig. 2: The authors emphasize that the Hill coefficient was much more sensitive than the activation and degradation parameters. It would be surprising if this was not the case, because the Hill coefficient is in the exponent of the equation. Indeed, Hill coefficients are notoriously difficult to fit.
3) The procedure shown in Fig. 3B is interesting, but some aspects are unclear. For example, the authors start with 3 free parameters. Which parameters were these? Was there a systemic testing of all possible parameter selections? Furthermore, which model was used for the simulations shown in Fig. 4? Was this performed with the model with 21 changed parameters? In Fig. 3B, 21 free parameters is the last bar. Would 24 changed parameters still improve the RMSE?
4) Fig. 5: Transcript and protein increases of STAT1, STAT2 and IRF9 upon IFN-beta stimulation are shown. However, model analysis and experiments were only performed on STAT2 and IRF9. At least model simulations should be performed on a STAT1 promoter mutant.
5) How were the cells analyzed in which the ISRE sites of IRF9 and STAT2 were removed via CRISPR-Cas9? Are these sites completely removed?

Minor comments:
1) On line 130, MLE-12 cells are introduced. It would be important to indicate that this is a mouse cell line.
2) The layout of Fig. 1E could be improved. It would probably help if the legend on top was in reverse order. In addition, maybe the color code of the cluster-specific GO terms could be repeated in the "Function" column.
3) Lines 176-177: The phases are described with overlapping time points. Furthermore, in the text all values are given in minutes, while in Fig. 2A the later time points are labeled with hours. It would be simpler to give ranges such as: 0 to 60 min, 80 min to 4 h, 6 to 12 h.
4) Is Supplementary Fig. 1 a part of the experiment shown in Fig. 2A? If yes, this should be indicated. Also, there is a typo in the y-axis of both panels.

Reviewer #2:

This is a very interesting paper that shows the dynamic responses to different IFN family members. the overall messages is that ISG are highly tunable. The work is well done and well presented. The authors should cite Yen et al., Cell 2022 that recently also eluded to some to the differential induction of anti-viral and proinflamamtory genes.

Reviewer #3:

The manuscript addresses an important and topical issue, i.e. the deciphering of the different responses of airway epithelial to one IFN-I subtype and one IFN-III subtype albeit both signaling pathways share ISFG3 as major transcription factor.

General statement:

The reviewer's field of expertise is not mathematical modeling hence the comments are only on the signaling and wet bench parts of the manuscript.

Unfortunately, the wet bench part shows some major flaws and open questions:
• in general, for the wet bench data more representative examples should be provided in the main Figures and the Supplemental Figures in order to allow the reader to get a feeling for the data quality.
• in particular Supplemental Fig 1 (legend is missing...) and the EMSA in Fig 2A is not convincing enough for the conclusions drawn and the further experiments and modeling built on. In addition to the general comment, Supplemental Fig 1 should also show similarities/discrepancies in the low dose-induced 'robust' (i.e. early antiviral) IFN response genes (e.g. DOI: 10.1126/scisignal.2004998) upon stimulus with IFNbeta and IFNlambda3. The data in Fig2A should be confirmed by further assessment of ISRE and GAS binding activities and pYSTAT1/2 at selected time points.
• in addition, the authors should ensure that the measured IFN-I and -III response differences do not simply origin in differences in pre-treatment equipment of the cells with IFNAR and IFNLR.
• the experimental setting grossly ignores describes stimulus-specific differences in IFN response counter regulatory mechanisms (e.g. DOI: 10.1074/jbc.M117.788877). SOCS1,3 and USP18 protein abundancies at selected times points should be provided for both stimuli.
• concerning the stimulus-contingent feed forward loop the authors describe highest positive autoregulation of IRF9 while STAT1 and STAT2 are less affected but behave similar. Nevertheless, further modeling and experimentation concentrated only on STAT2 and IRF9, not STAT1. This rationale for this decision should be made clear.
• IFN-repressed genes (IRepG) have become an established class among the IFN response genes (e.g. DOI: 0.1093/nar/gkt589; DOI: 10.3389/fimmu.2017.01139); are IRepG observed in a stimulus- and/or time course-dependent manner?

Reviewer #1:

In this manuscript, Wilder and coworkers investigate differential gene responses induced by interferon (IFN)-beta vs. IFN-lambda. Experiments were performed in mouse lung epithelial cells (MLE-12). Time-resolved RNA-seq data showed that IFN-beta induced a strong, transient induction 4 to 8 h after stimulation of a large cluster of genes associated with cytokine production and immune response. The same genes showed a sustained accumulation with slower dynamics upon stimulation with IFN-lambda. Similar dynamics were observed by an electrophoretic mobility shift assay (EMSA) using an ISRE probe. A simple mathematical model demonstrated that the average expression profile upon IFN-beta and IFN-lambda stimulation could be explained by this ISRE binding data. A previously published mathematical model was re-fitted to time-resolved experimental data based on immunoblotting and EMSA experiments. Model simulations and experiments showed that for the high and transient gene expression, a high and sustained dose of IFN-beta as well as de novo protein synthesis was required. IFN-beta induced the expression of IRF9 and STAT2, comprising a positive feedback loop. By removing the ISRE sites of IRF9 and STAT2 via CRISPR-Cas9, this positive feedback loop was abolished and consequently the high and transient gene expression induced by IFN-beta was reduced.
The experimental data as well as the mathematical modeling approaches are of high quality. The conclusions, although not completely unexpected, are supported by the data and clearly described. Dynamic control of IFN-induced gene response is a timely topic, given the importance of IFN signal transduction in antiviral responses.

We thank the reviewer for their appreciative comments.
Major comments:
1) Fig. 1D: The selection of genes was based on transcripts that are induced by IFN-beta. Are there transcripts that are only induced by IFN-lambda? If yes, a supplementary figure with these transcripts should be included.
A small subset of 27 genes was categorized IFN-lambda-specific. We included these genes in the supplement.

2) Fig. 2: The authors emphasize that the Hill coefficient was much more sensitive than the activation and degradation parameters. It would be surprising if this was not the case, because the Hill coefficient is in the exponent of the equation. Indeed, Hill coefficients are notoriously difficult to fit.
We agree with the reviewer that because the Hill coefficient is in the exponent it can have a large effect. In our case, because the data is max-normalized, the Hill coefficient only affects the dose-response curve.

3) The procedure shown in Fig. 3B is interesting, but some aspects are unclear. For example, the authors start with 3 free parameters. Which parameters were these? Was there a systemic testing of all possible parameter selections? Furthermore, which model was used for the simulations shown in Fig. 4? Was this performed with the model with 21 changed parameters? In Fig. 3B, 21 free parameters is the last bar. Would 24 changed parameters still improve the RMSE?
We apologize that not all of this was clearly described. The parameters chosen for each model simulation was indicated in the Supplemental Table 3. The order of parameters chosen was based on which would provide the best fit. The 21 param free model was indeed used for the model simulations for the remainder of the manuscript. The aim for the model fitting was to make the minimal number of necessary changes to the published model to obtain an optimal fit while also guarding against overfitting. Since the change in error had begun to level after fitting 21 parameters, we chose the 21-free-parameter model. We have added the error when all the parameters are fitted concurrently to make this point clear. We have also added additional text to clarify and clearly state all of this.

4) Fig. 5: Transcript and protein increases of STAT1, STAT2 and IRF9 upon IFN-beta stimulation are shown. However, model analysis and experiments were only performed on STAT2 and IRF9. At least model simulations should be performed on a STAT1 promoter mutant.
Thank you for this suggestion. Experiments were carried out on the two top viable candidates, STAT2 and IRF9, which are also less promiscuous in forming dimers with other complexes (i.e. GAF) compared to the ISGF3 heterodimer. Model simulations for the STAT1 promoter mutant were performed and included in the supplement.

5) How were the cells analyzed in which the ISRE sites of IRF9 and STAT2 were removed via CRISPR-Cas9? Are these sites completely removed?
Sanger sequencing and amplicon sequencing was used to confirm the successful mutation at the ISRE sites. This data has been included as a supplemental figure.

Minor comments:
1) On line 130, MLE-12 cells are introduced. It would be important to indicate that this is a mouse cell line.
We apologize for this oversight, and have now indicated this.

2) The layout of Fig. 1E could be improved. It would probably help if the legend on top was in reverse order. In addition, maybe the color code of the cluster-specific GO terms could be repeated in the "Function" column.
Thank you for this suggestion. Changes were made to improve this figure.
3) Lines 176-177: The phases are described with overlapping time points. Furthermore, in the text all values are given in minutes, while in Fig. 2A the later time points are labeled with hours. It would be simpler to give ranges such as: 0 to 60 min, 80 min to 4 h, 6 to 12 h.
We can improve this and changes have been made in the text to match the labeling in the figure.
4) Is Supplementary Fig. 1 a part of the experiment shown in Fig. 2A? If yes, this should be indicated. Also, there is a typo in the y-axis of both panels.
Thank you for this point. We have added text to that indicates this data is a subset of that from Fig 2 and corrected the y-axis label.

Reviewer #2:

This is a very interesting paper that shows the dynamic responses to different IFN family members. the overall messages is that ISG are highly tunable. The work is well done and well presented. The authors should cite Yen et al., Cell 2022 that recently also eluded to some to the differential induction of anti-viral and proinflamamtory genes.
We thank the reviewer for their appreciative comments. Thanks for pointing out the very interesting paper, which shows (Fig.5) that engineered interferon variants can have differential innate immune vs inflammatory effects. The present manuscript addresses some of the potential underlying mechanisms. We have now cited in the revision.

Reviewer #3:

The manuscript addresses an important and topical issue, i.e. the deciphering of the different responses of airway epithelial to one IFN-I subtype and one IFN-III subtype albeit both signaling pathways share ISFG3 as major transcription factor.

General statement:
The reviewer's field of expertise is not mathematical modeling hence the comments are only on the signaling and wet bench parts of the manuscript.

Unfortunately, the wet bench part shows some major flaws and open questions:
• in general, for the wet bench data more representative examples should be provided in the main Figures and the Supplemental Figures in order to allow the reader to get a feeling for the data quality.
Thank you for this suggestion. Images of the nuclear and cytoplasmic IFN signaling proteins (immunoblots) and ISGF3 DNA binding activity (EMSA) data has been added as Extended Data.

• in particular Supplemental Fig 1 (legend is missing...) and the EMSA in Fig 2A is not convincing enough for the conclusions drawn and the further experiments and modeling built on. In addition to the general comment, Supplemental Fig 1 should also show similarities/discrepancies in the low dose-induced 'robust' (i.e. early antiviral) IFN response genes (e.g. DOI: 10.1126/scisignal.2004998) upon stimulus with IFNbeta and IFNlambda3. The data in Fig2A should be confirmed by further assessment of ISRE and GAS binding activities and pYSTAT1/2 at selected time points.
Thank you for pointing out the missing legend. We have now included a legend for Supplemental Fig.1. We also appreciate the reviewer's comment about Fig.2A. We apologize that the reasons for our conclusions were not clear. In the manuscript, we experimentally confirmed the IFN-specific temporal dynamics of ISGF3 in Fig.2A using perturbation conditions (i.e. low dose, pulse, cycloheximide, positive feedback mutants) using the ISRE mobility shift assays as seen in Fig.4C-F, Fig.5A, and Fig.5F. The data for these line plots has been included as extended data. Since the scope of the latter half of the manuscript was centered on the mechanisms controlling the IFN-beta-induced potentiated phase of ISGF3, only perturbations of IFN-beta conditions were included. In the manuscript, the measurements of nuclear and cytoplasmic pYSTAT1/2 data were included in Fig.3C, but we have added the data (immunoblots) in the extended data figures. In this study, the robust IFN response genes were found among the cluster 2 (i.e. IFN common) genes. Examples of robust IFN response genes are shown in Fig.1F among the cluster 2 genes. Many of these genes (e.g. IRF7, RSAD2, TLR3, DDX58, and IFI35) were also classified as robust genes in the indicated article (DOI: 10.1126/scisignal.2004998). We have now added text indicating these genes as previously identified robust IFN response genes and referenced this article.

• in addition, the authors should ensure that the measured IFN-I and -III response differences do not simply origin in differences in pre-treatment equipment of the cells with IFNAR and IFNLR.
This is a great point, and we thank the reviewer for this caution. We acknowledge that differences in receptor expression can affect ISGF3 activation. Due to this, the amount of IFN-beta and IFN-lambda used was chosen to

ensure that the number of receptors engaged caused similar initial pathway activation (Supplemental Fig 1). Thus, any differences seen in gene expression are due to differential signaling or gene regulatory mechanisms. Additional text has been added to make this point clear.

• the experimental setting grossly ignores describes stimulus-specific differences in IFN response counter regulatory mechanisms (e.g. DOI: 10.1074/jbc.M117.788877). SOCS1,3 and USP18 protein abundancies at selected times points should be provided for both stimuli.
We appreciate this comment and agree with the reviewer that there may be stimulus-specific differences in the negative regulatory mechanisms, but this was not the focus of the manuscript since the phenomenon discovered (i.e. ISGF3 potentiated phase) would require activation from a positive regulator. Since the negative regulators do not provide an explanation for the phenomenon under study we did not include any of that analysis in our focus. We did include additional text in the Discussion section to bring up this point.

• concerning the stimulus-contingent feed forward loop the authors describe highest positive autoregulation of IRF9 while STAT1 and STAT2 are less affected but behave similar. Nevertheless, further modeling and experimentation concentrated only on STAT2 and IRF9, not STAT1. This rationale for this decision should be made clear.
Thank you for this suggestion. The rationale for this is that STAT1 is also involved in GAF binding to control expression of other genes. Experiments were carried out on the two top viable candidates, STAT2 and IRF9, which are also less promiscuous in forming dimers with other complexes (i.e. GAF) compared to the ISGF3 heterodimer. Additional text has been added to make this clear.

• IFN-repressed genes (IRepG) have become an established class among the IFN response genes (e.g. DOI: 0.1093/nar/gkt589; DOI: 10.3389/fimmu.2017.01139); are IRepG observed in a stimulus- and/or time course-dependent manner?
IFN-repressed genes do not provide an explanation for the phenomenon (i.e. ISGF3 potentiated phase) under study. Hence, they are not the focus of this manuscript. Initial analysis of the RNAseq data also did not yield a repressed gene expression program. We did include additional text in the discussion section to bring up this point.

Thank you for sending us your revised manuscript. We have now heard back from the two reviewers who were asked to evaluate your revised study. As you will see below, both reviewers are satisfied with the performed revisions and support publication.

Before we formally accept the manuscript for publication, we would ask you to address some remaining editorial issues listed below.

Reviewer #1:

The authors have adequately and convincingly responded to all issues raised. The manuscript has been significantly improved.

Reviewer #3:

The authors have addressed the concerns and queries of the referee in an adequate and satisfactory manner.
In addition, I was asked to assess the answers to the points raised by the other referees. To my opinion, also these queries have been appropriately addressed.
The manuscript is now suitable for publication.

All editorial and formatting issues were resolved by the authors.

2nd Revision - Editorial Decision **1st Mar 2023**

Thank you again for sending us your revised manuscript. We are now satisfied with the modifications made and I am pleased to inform you that your paper has been accepted for publication.

EMBO Press Author Checklist

Corresponding Author Name: Alexander Hoffmann	
Journal Submitted to: MSB	
Manuscript Number: MSB-2022-11294R	

USEFUL LINKS FOR COMPLETING THIS FORM
The EMBO Journal - Author Guidelines
EMBO Reports - Author Guidelines
Molecular Systems Biology - Author Guidelines
EMBO Molecular Medicine - Author Guidelines

Reporting Checklist for Life Science Articles (updated January
This checklist is adapted from Materials Design Analysis Reporting (MDAR) Checklist for Authors. MDAR establishes a minimum set of requirements in transparent reporting in the life sciences (see Statement of Task: 10.31222/osf.io/9sm4x). Please follow the journal's guidelines in preparing your
Please note that a copy of this checklist will be published alongside your article.

Abridged guidelines for figures
1. Data
The data shown in figures should satisfy the following conditions:
- the data were obtained and processed according to the field’s best practice and are presented to reflect the results of the experiments in an accurate and unbiased manner.
- ideally, figure panels should include only measurements that are directly comparable to each other and obtained with the same assay.
- plots include clearly labeled error bars for independent experiments and sample sizes. Unless justified, error bars should not be shown for technical
- if n<5, the individual data points from each experiment should be plotted. Any statistical test employed should be justified.
- Source Data should be included to report the data underlying figures according to the guidelines set out in the authorship guidelines on Data

2. Captions
Each figure caption should contain the following information, for each panel where they are relevant:
- a specification of the experimental system investigated (eg cell line, species name).
- the assay(s) and method(s) used to carry out the reported observations and measurements.
- an explicit mention of the biological and chemical entity(ies) that are being measured.
- an explicit mention of the biological and chemical entity(ies) that are altered/varied/perturbed in a controlled manner.
- the exact sample size (n) for each experimental group/condition, given as a number, not a range;
- a description of the sample collection allowing the reader to understand whether the samples represent technical or biological replicates (including how many animals, litters, cultures, etc.).
- a statement of how many times the experiment shown was independently replicated in the laboratory.
- definitions of statistical methods and measures:
  - common tests, such as t-test (please specify whether paired vs. unpaired), simple χ2 tests, Wilcoxon and Mann-Whitney tests, can be unambiguously identified by name only, but more complex techniques should be described in the methods section;
  - are tests one-sided or two-sided?
  - are there adjustments for multiple comparisons?
  - exact statistical test results, e.g., P values = x but not P values < x;
  - definition of ‘center values’ as median or average;
  - definition of error bars as s.d. or s.e.m.

Please complete ALL of the questions below.
Select "Not Applicable" only when the requested information is not relevant for your study.

Materials

Newly Created Materials	Information included in the manuscript?	In which section is the information available? (Reagents and Tools Table, Materials and Methods, Figures, Data Availability Section)
New materials and reagents need to be available; do any restrictions apply?	Yes	

Antibodies	Information included in the manuscript?	In which section is the information available? (Reagents and Tools Table, Materials and Methods, Figures, Data Availability Section)
For **antibodies** provide the following information:<br>- Commercial antibodies: RRID (if possible) or supplier name, catalogue number and or/clone number<br>- Non-commercial: RRID or citation	Yes	

DNA and RNA sequences	Information included in the manuscript?	In which section is the information available? (Reagents and Tools Table, Materials and Methods, Figures, Data Availability Section)
**Short novel DNA or RNA including primers, probes**: provide the sequences.	Yes	

Cell materials	Information included in the manuscript?	In which section is the information available? (Reagents and Tools Table, Materials and Methods, Figures, Data Availability Section)
**Cell lines:** Provide species information, strain. Provide accession number in repository **OR** supplier name, catalog number, clone number, and/**OR** RRID.	Yes	
**Primary cultures:** Provide species, strain, sex of origin, genetic modification status.	Not Applicable	
Report if the cell lines were recently **authenticated** (e.g., by STR profiling) and tested for mycoplasma contamination.	Yes	

Experimental animals	Information included in the manuscript?	In which section is the information available? (Reagents and Tools Table, Materials and Methods, Figures, Data Availability Section)
**Laboratory animals or Model organisms:** Provide species, strain, sex, age, genetic modification status. Provide accession number in repository **OR** supplier name, catalog number, clone number, **OR** RRID.	Not Applicable	
**Animal observed in or captured from the field:** Provide species, sex, and age where possible.	Not Applicable	
Please detail **housing and husbandry conditions**.	Not Applicable	

Plants and microbes	Information included in the manuscript?	In which section is the information available? (Reagents and Tools Table, Materials and Methods, Figures, Data Availability Section)
**Plants:** provide species and strain, ecotype and cultivar where relevant, unique accession number if available, and source (including location for collected wild specimens).	Not Applicable	
**Microbes:** provide species and strain, unique accession number if available, and source.	Not Applicable	

Human research participants	Information included in the manuscript?	In which section is the information available? (Reagents and Tools Table, Materials and Methods, Figures, Data Availability Section)
If collected and within the bounds of privacy constraints report on age, sex and gender or ethnicity for all study participants.	Not Applicable	

Core facilities	Information included in the manuscript?	In which section is the information available? (Reagents and Tools Table, Materials and Methods, Figures, Data Availability Section)
If your work benefited from core facilities, was their service mentioned in the acknowledgments section?	Yes	

Design

Study protocol	Information included in the manuscript?	In which section is the information available? (Reagents and Tools Table, Materials and Methods, Figures, Data Availability Section)
If study protocol has been **pre-registered, provide DOI in the manuscript**. For clinical trials, provide the trial registration number **OR** cite DOI.	Not Applicable	
Report the **clinical trial registration number** (at ClinicalTrials.gov or equivalent), where applicable.	Not Applicable	

Laboratory protocol	Information included in the manuscript?	In which section is the information available? (Reagents and Tools Table, Materials and Methods, Figures, Data Availability Section)
Provide DOI OR other citation details if **external detailed step-by-step protocols** are available.	Not Applicable	

Experimental study design and statistics	Information included in the manuscript?	In which section is the information available? (Reagents and Tools Table, Materials and Methods, Figures, Data Availability Section)
Include a statement about **sample size** estimate even if no statistical methods were used.	Yes	
Were any steps taken to minimize the effects of subjective bias when allocating animals/samples to treatment (e.g. **randomization procedure**)? If yes, have they been described?	Not Applicable	
Include a statement about **blinding** even if no blinding was done.	Not Applicable	
Describe **inclusion/exclusion criteria** if samples or animals were excluded from the analysis. Were the criteria pre-established?<br><br>If sample or data points were omitted from analysis, report if this was due to attrition or intentional exclusion and provide justification.	Not Applicable	
For every figure, are **statistical tests** justified as appropriate? Do the data meet the assumptions of the tests (e.g., normal distribution)? Describe any methods used to assess it. Is there an estimate of variation within each group of data? Is the variance similar between the groups that are being statistically compared?	Yes	

Sample definition and in-laboratory replication	Information included in the manuscript?	In which section is the information available? (Reagents and Tools Table, Materials and Methods, Figures, Data Availability Section)
In the figure legends: state number of times the experiment was **replicated** in laboratory.	Yes	
In the figure legends: define whether data describe **technical or biological replicates**.	Yes	

Ethics

Ethics	Information included in the manuscript?	In which section is the information available? (Reagents and Tools Table, Materials and Methods, Figures, Data Availability Section)
Studies involving **human participants**: State details of **authority granting ethics approval** (IRB or equivalent committee(s), provide reference number for approval.	Not Applicable	
Studies involving **human participants**: Include a statement confirming that **informed consent** was obtained from all subjects and that the experiments conformed to the principles set out in the WMA Declaration of Helsinki and the Department of Health and Human Services Belmont Report.	Not Applicable	
Studies involving **human participants:** For publication of **patient photos**, include a statement confirming that consent to publish was obtained.	Not Applicable	
Studies involving experimental **animals**: State details of **authority granting ethics approval** (IRB or equivalent committee(s), provide reference number for approval. Include a statement of compliance with ethical regulations.	Not Applicable	
Studies involving **specimen and field samples:** State if relevant **permits** obtained, provide details of authority approving study; if none were required, explain why.	Not Applicable	

Dual Use Research of Concern (DURC)	Information included in the manuscript?	In which section is the information available? (Reagents and Tools Table, Materials and Methods, Figures, Data Availability Section)
Could your study fall under dual use research restrictions? Please check biosecurity documents and list of **select agents and toxins** (CDC): https://www.selectagents.gov/sat/list.htm	Not Applicable	
If you used a select agent, is the security level of the lab appropriate and reported in the manuscript?	Not Applicable	
If a study is subject to dual use research of concern regulations, is the name of the **authority granting approval and reference number** for the regulatory approval provided in the manuscript?	Not Applicable	

Reporting

The MDAR framework recommends adoption of discipline-specific guidelines, established and endorsed through community initiatives. Journals have their own policy about requiring specific guidelines and recommendations to complement MDAR.

Adherence to community standards	Information included in the manuscript?	In which section is the information available? (Reagents and Tools Table, Materials and Methods, Figures, Data Availability Section)
State if relevant guidelines or checklists (e.g., **ICMJE, MIBBI, ARRIVE, PRISMA**) have been followed or provided.	Not Applicable	
For **tumor marker prognostic studies,** we recommend that you follow the **REMARK** reporting guidelines (see link list at top right). See author guidelines, under 'Reporting Guidelines'. Please confirm you have followed these guidelines.	Not Applicable	
For **phase II and III randomized controlled trials**, please refer to the **CONSORT** flow diagram (see link list at top right) and submit the CONSORT checklist (see link list at top right) with your submission. See author guidelines, under 'Reporting Guidelines'. Please confirm you have submitted this list.	Not Applicable	

Data Availability

Data availability	Information included in the manuscript?	In which section is the information available? (Reagents and Tools Table, Materials and Methods, Figures, Data Availability Section)
Have **primary datasets** been deposited according to the journal's guidelines (see 'Data Deposition' section) and the respective accession numbers provided in the Data Availability Section?	Yes	
Were **human clinical and genomic datasets** deposited in a public access-controlled repository in accordance to ethical obligations to the patients and to the applicable consent agreement?	Not Applicable	
Are **computational models** that are central and integral to a study available without restrictions in a machine-readable form? Were the relevant accession numbers or links provided?	Yes	
If publicly available data were reused, provide the respective **data citations in the reference list**.	Yes	
